# Core profile of volatile organic compounds related to growth of *Mycobacterium avium* subspecies *paratuberculosis* – A comparative extract of three independent studies

**DOI:** 10.1371/journal.pone.0221031

**Published:** 2019-08-15

**Authors:** Anne Küntzel, Michael Weber, Peter Gierschner, Phillip Trefz, Wolfram Miekisch, Jochen K. Schubert, Petra Reinhold, Heike Köhler

**Affiliations:** 1 Institute of Molecular Pathogenesis, Friedrich-Loeffler-Institut (FLI), Federal Research Institute for Animal Health, Jena, Germany; 2 Rostock Medical Breath Research Analytics and Technologies (RoMBAT), Department of Anaesthesia and Intensive Care, Rostock University Medical Center, Rostock, Germany; 3 National Reference Laboratory for Paratuberculosis, FLI, Jena, Germany; Cornell University, UNITED STATES

## Abstract

Analysis of volatile organic compounds (VOC) derived from bacterial metabolism during cultivation is considered an innovative approach to accelerate *in vitro* detection of slowly growing bacteria. This applies also to *Mycobacterium avium* subsp. *paratuberculosis* (MAP), the causative agent of paratuberculosis, a debilitating chronic enteritis of ruminants. Diagnostic application demands robust VOC profiles that are reproducible under variable culture conditions. In this study, the VOC patterns of pure bacterial cultures, derived from three independent *in vitro* studies performed previously, were comparatively analyzed. Different statistical analyses were linked to extract the VOC core profile of MAP and to prove its robustness, which is a prerequisite for further development towards diagnostic application. Despite methodical variability of bacterial cultivation and sample pre-extraction, a common profile of 28 VOCs indicating cultural growth of MAP was defined. The substances cover six chemical classes. Four of the substances decreased above MAP and 24 increased. Random forest classification was applied to rank the compounds relative to their importance and for classification of MAP versus control samples. Already the top-ranked compound alone achieved high discrimination (AUC 0.85), which was further increased utilizing all compounds of the VOC core profile of MAP (AUC 0.91). The discriminatory power of this tool for the characterization of natural diagnostic samples, in particular its diagnostic specificity for MAP, has to be confirmed in future studies.

## Introduction

*Mycobacterium avium* ssp. *paratuberculosis* (MAP) causes chronic granulomatous enteritis in ruminants. Infected animals shed the organism already months to years before clinical signs become evident and thus, contribute to unrecognized spread of the disease. Paratuberculosis, or Johne’s disease, affects all domestic and wild ruminants and is spread worldwide. Due to the lack of effective therapeutics for ruminants, infected animals need to be detected and removed from the herd as early as possible. Therefore, a sensitive, rapid and cost-efficient diagnostic method identifying ideally even sub-clinically infected animals is urgently required. Currently, the most sensitive diagnostic method is cultural isolation of MAP on solid or liquid media with subsequent species confirmation via polymerase chain reaction [1, 2], i.e. a labor-intensive, expensive, and very time-consuming procedure taking weeks to months until a reliable result is available [3, 4]. Automated liquid culture systems, which were adopted recently for MAP, resulted in reduced cultivation times but still demand further processing of the samples for species identification. The underlying detection principle is based on non-specific parameters like gas consumption by viable mycobacteria which creates a negative pressure change in the headspace above the broth culture medium or a fluorescent indicator which reacts to alterations in oxygen, CO_2_ and pressure inside the culture tubes [5–8].

Detection of volatile organic compounds (VOC) derived from bacterial metabolism has been proposed as a novel approach in diagnostic microbiology [9], and putative species-specific VOC-profiles detected in the headspace of bacterial cultures have been published [9–12]. Online monitoring of bacteria-specific VOC-profiles would enable direct species identification without further processing of samples. Consequentially, VOC analysis offers the potential of a modern, innovative, and rapid diagnostic tool detecting cultural growth of MAP *in vitro*.

The data published on bacteria-related VOC analyses are still of descriptive character. Due to methodological variation of bacterial cultivation and/or VOC measurement, no robust and reproducible VOC-profile has been documented so far for any bacterial species. Qualitative or quantitative methodical factors contributing to the variability of the VOC-profiles, such as preparation of the inoculum, quality of medium used for bacterial culture, temperature of incubation, incubation time, and bacterial density have been reported in several studies (Table 1).

**Table 1:** Methodical factors influencing the VOC-profile during bacterial cultivation.

Influencing factor	References	Pathogen
Medium composition	Ratiu et al. 2017 [13]	*Escherichia coli*
	Küntzel et al. 2016 [14]	MAP
	Nawrath et al. 2012 [15]	*M*. *tuberculosis*, non-tuberculous mycobacteria, *Nocardia* spp.
	O'Hara and Mayhew 2009 [16]	*Staphylococcus aureus*
	Scotter et al. 2005 [17]	*Aspergillus flavus*, *Aspergillus fumigatus*, *Candida albicans*, *Mucor racemosus*, *Fusarium solani*, *Cryptococcus neoformans*
Duration of incubation	Küntzel et al. 2016 [14]	MAP
	Rees et al. 2016 [18]	*Klebsiella pneumoniae*
	Nawrath et al. 2012 [15]	*M*. *tuberculosis*, non-tuberculous mycobacteria, *Nocardia* spp.
	O'Hara and Mayhew 2009 [16]	*Staphylococcus aureus*
	Bunge et al. 2008 [10]	*Escherichia coli*, *Shigella flexneri*, *Salmonella enterica*, *Candida tropicalis*
Bacterial density	Küntzel et al. 2016 [14]	MAP
	Trefz et al. 2013 [19]	MAP
Temperature	Küntzel et al. 2018 [20]	*Mycobacteria* (different species)
Oxygen supply	Rees et al. 2017 [21]	*Aspergillus fumigatus*

MAP: *Mycobacterium avium* ssp. *paratuberculosis*

With respect to MAP cultivation, three independent *in vitro* studies performed previously resulted in heterogeneous VOC patterns although the following variables were kept constant: medium used for bacterial cultivation, incubation temperature, and a defined bacterial density of the inoculum [14, 19, 20]. Bacterial heterogeneity was ensured by including six different MAP strains. The growth of bacteria over time was unpredictable and was considered a variable factor. However, several VOCs were identified in all three studies and the question arose whether a VOC core profile of MAP could be extracted from the results of these studies. A VOC core profile is supposed to be a reproducible restricted group/pattern of VOCs identifiable and quantifiable in samples containing MAP independent from methodical and biological variability.

In the present study, different statistical analyses were linked to extract the VOC core profile of MAP and to prove its robustness, which is a prerequisite for further development towards diagnostic application.

## Methods

Data of three independent studies performed in different years, by different experimenters and with different experimental set-ups were analyzed [14, 19, 20]. In total, five field isolates and one reference strain of MAP were included in these studies. In addition, 15 strains of Mycobacteria belonging to 12 species different from MAP were included in Study 3 [20]. In all three studies, preparation and cultivation of bacteria followed a defined protocol in order to provide similar and comparable conditions for bacterial growth and VOC headspace sampling. For comparison, Table 2 summarizes the main methodological aspects of the three studies.

**Table 2:** Design of the three studies taken into account for comparative exploitation.

	Study 1		Study 2		Study 3	
	[19]		[14]		[20]	
**Carried out in**	2011		2012		2016	
**MAP strains**	1	MAP DSM-44133 (type II, DSMZ)	A	MAP DSM-44133 (type II, DSMZ)	A	MAP DSM-44133 (type II, DSMZ)
	2	MAP 04A0386 (type III, field isolate from sheep)	B	MAP 04A0386 (type III, field isolate from sheep)	B	MAP 04A0386 (type III, field isolate from sheep)
	3	MAP 05A2421 (type II, field isolate from cattle)	C	MAP 12MA1245 (type II, field isolate from cattle)		
	4	MAP 05A3197 (type II, field isolate from cattle)				
	5	MAP 06A0817 (type II, field isolate from red deer)				
**Medium**	Herrold’s Egg Yolk Medium with MJ		Herrold’s Egg Yolk Medium with MJ		Herrold’s Egg Yolk Medium with MJ	
**Incubation temperature**	37°C		37°C		37°C	
**Incubation period**	6 weeks		2, 4 and 6 weeks		4 weeks	
**Inoculum**	Original suspension (OD: n.d.) and dilutions of 10^−2^, 10^−4^ and 10^−6^		Original suspension (OD: 0.316±0.015) and dilutions of 10^−2^, 10^−4^ and 10^−6^		Original suspension (OD: 0.306±0.02)	
**Pre-concentration**	Solid phase micro extraction		Needle trap micro extraction		Needle trap micro extraction	
**VOC Analysis**	GC-MS		GC-MS		GC-MS	
**Substance identification**	NIST 2005 Gatesburg and analysis of pure reference substances		NIST 2005 Gatesburg and analysis of pure reference substances		NIST 2005 Gatesburg and analysis of pure reference substances	

MAP: *Mycobacterium avium* ssp. *paratuberculosis*, DSMZ: German Collection of Microorganisms and Cell Cultures, MJ: Mycobactine J, OD: optical density, GC-MS: gas chromatography–mass spectrometry, n.d.: not defined

### Intersection analysis

In each study a MAP-related VOC-profile was defined applying the following selection criteria:

- The VOC-profile was defined at the time point of exponential bacterial growth.
- The concentration of the compounds in the laboratory room air needed to be significantly lower than in the headspace of the vials.
- VOC concentration above MAP and above control vials needed to differ significantly (lower or higher).

**Fig 1:**
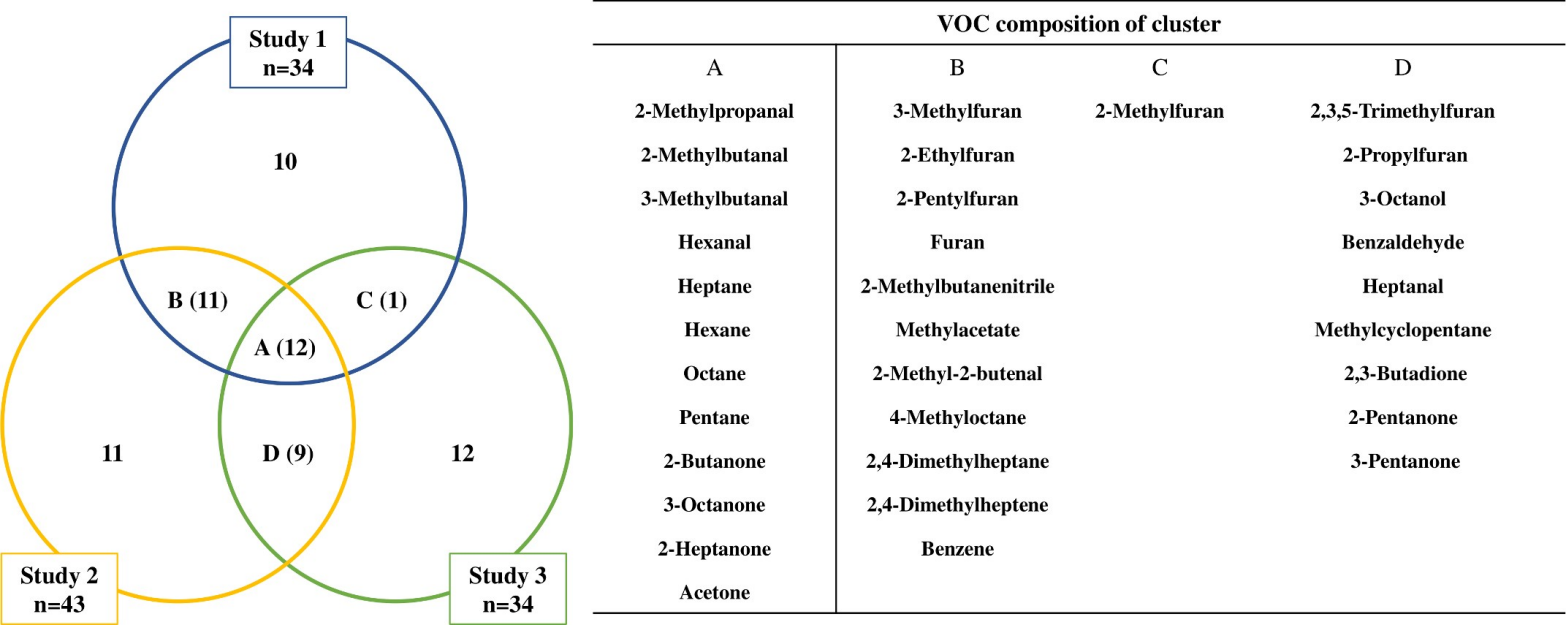
Overlap of MAP-related VOCs identified in the three studies. Left: Number of overlapping VOCs in all three studies (cluster A) and in two of the studies (clusters B-D). Right: VOCs contained in clusters A-D.

This resulted in MAP-related VOC-profiles including 34 substances in Study 1 and Study 3, respectively, and 43 substances in Study 2 (Fig 1).

Intersection analysis of the results of the three studies included the following steps:

1. Identification of VOCs that differed significantly between inoculated and control vials in at least two of the studies.
2. Selection of compounds that followed a reproducible trend in concentration, i.e. decreasing or increasing compared to pure medium.
3. Quantitative assessment of the relative change compared to pure medium.

Concentration values of selected VOCs of all control vials (Study 1: n = 2, Study 2 n = 10, Study 3: n = 6) and inoculated vials (Study 1: n = 40, Study 2: n = 23, Study 3: n = 6) were included in the data analysis. Microsoft Excel 2016 (Microsoft Corporation, USA) was used to normalize VOC-concentrations above MAP to the median concentration of control vials of each study for comparing the relative change across the three studies. Further statistical calculations were performed in the statistical environment R.

### Meta-statistics on pooled data

Statistical analysis of pooled data of all three studies was performed subsequently to confirm the significance of the findings. This meta-statistics comprised pooling, scaling and testing of the present data with the advantage of increased sample sizes compared to individual study tests. All VOCs with measurement data for at least two studies were selected for integrated analysis (n = 39, S1 Table).

Measurement values from the different studies showed heterogeneous numerical ranges and needed to be standardized. The data were standardized by dividing each value by the maximum value for the given substance and experiment. As a result, standardized substance measurements ranged between 0 and 1 and could be pooled into two general groups (inoculated and control). In the next step, statistical tests were applied to identify significant differences between both groups. Differences in the VOC abundance between inoculated and control samples were analyzed by applying the non-parametric Mann-Whitney U-test for each substance. The resulting p-values were further adjusted for multiple testing with the Benjamini-Hochberg FDR method. All substances with an adjusted p-value < 0.01 were regarded significantly different.

#### Classification with Random forest

The classification method Random forest was applied for computation of feature importance and classification of MAP versus control samples. The first step involved application of the feature selection algorithm Boruta, which utilizes Random forest variable importance to calculate scores on original data compared to a randomly permuted copy [22]. The resulting median importance was used to order VOCs from highest to lowest importance.

Next, classification of the samples was performed to distinguish MAP versus control group. Therefore, the R package caret was applied which depends on the package RandomForest [23]. Parameters of the model (mtry and ntree) are tuned by the caret method.

To obtain reasonable estimates of the classification accuracy, cross-validation was performed on all possible pairs of the three data sets (n = 6). For each pair, the first set was used for training of the classifier, while the second set was used to estimate the classification accuracy—sensitivity and specificity. Furthermore, care was taken that each data set contained the same number of MAP-positive and control samples. Consequentially, up-sampling was performed, i.e. resampling with replacement in order to balance the number of samples in both groups. As a classification result, ROC-curves were generated, which summarize all classification runs and estimate classification performance for all available data. Curves were plotted with the plotROC package [24].

## Results

Taking the results of all three studies into account, more than 100 VOCs were measureable in the headspace of the vials. In total, 66 substances were significant for MAP in at least one of the studies. Comparing the outcome of the three studies concerning MAP-related VOCs for overlapping results, 12 substances were identified (Fig 1A). Additional 21 substances were present in only two studies (Fig 1B–1D).

**Fig 2:**
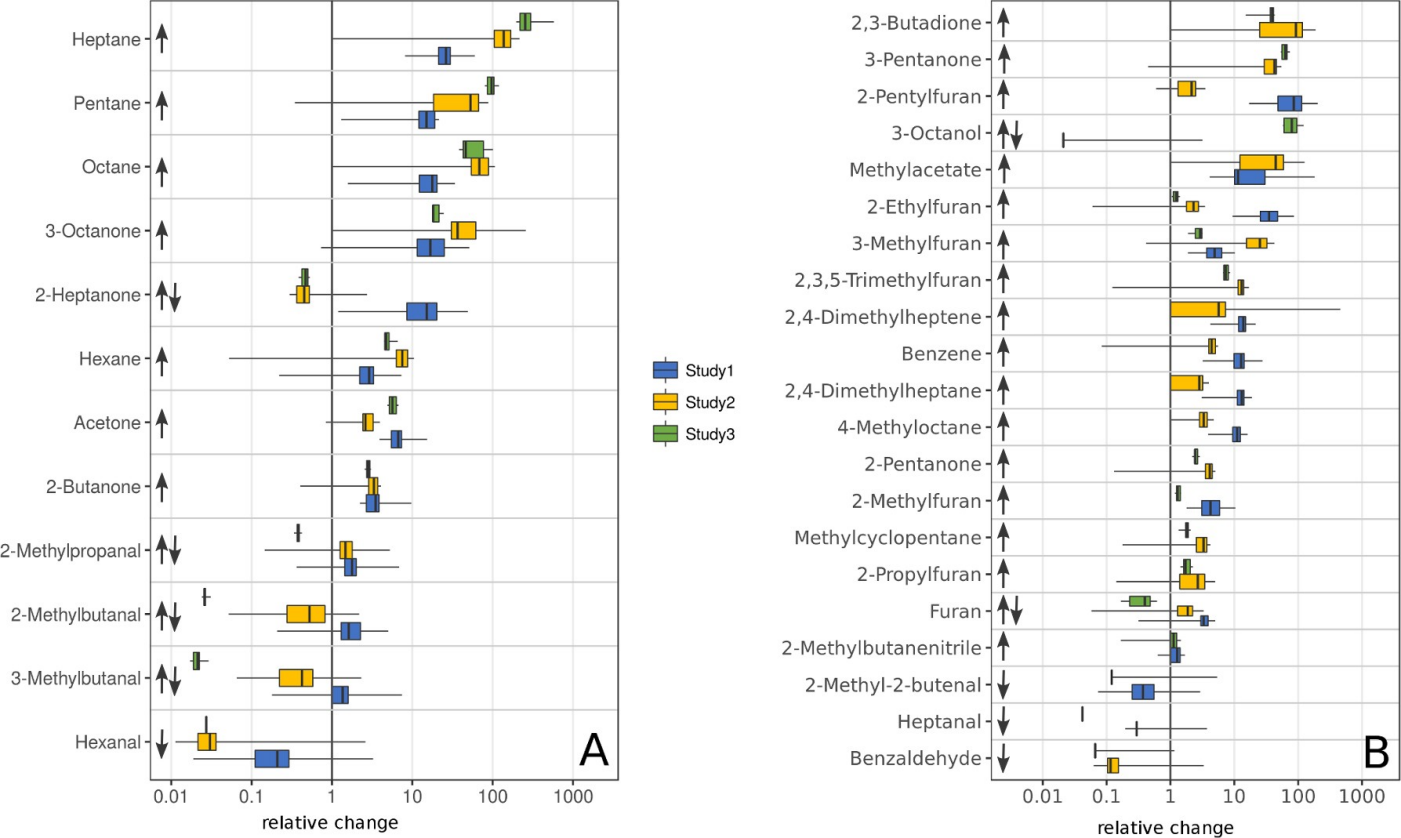
VOC concentration levels of all substances per study normalized to the median concentration of their respective pure medium. Values below 1 indicate a decreasing VOC concentration above MAP (↓) and values higher than 1 indicate an increasing VOC concentration above MAP (↑). A: Substances being significant for MAP in all three studies. B: Substances being significant for MAP in two of three studies. Study 1: MAP cultures n = 40, control vials n = 2, Study 2: MAP cultures n = 23, control vials n = 10, Study 3: MAP cultures n = 6, control vials n = 6.

The VOC concentrations of 27 out of these 33 substances showed the same tendencies above growing bacteria in comparison to control vials. Twenty-three volatiles increased and four decreased above MAP in all of the studies where data was available. Furan, 2-Methylpropanal, 2-Methylbutanal, 2-Heptanone, 3-Methylbutanal, and 3-Octanol showed differing tendencies across the three studies (Fig 2 ↑↓).

Total values of VOC concentrations above pure media differed significantly amongst the three studies and therefore VOC concentrations above MAP differed significantly as well (S1 Table). By matching VOC concentrations above bacteria to median values of measurements above control vials of each study, the magnitude of relative change across the three studies could be compared (Fig 2).

For 18 substances the medians of the relative change were in the same range of values of all available studies (S2 Table, bold writing). For 7 of those 18 this was true for all three studies (Octane, Pentane, 3-Octanone, Hexane, 2-Butanone, Acetone and Hexanal).

Based on the intersection approach, a profile of 27 substances indicating MAP growth was identified, 18 of which showed similar directions and magnitudes of change (increase or decrease) amongst all available data (S2 Table).

By taking all VOCs with available measurement data for at least two studies into account, meta-statistics resulted in 30 substances being significant for MAP in comparison to control vials (Table 3).

**Table 3:** Results of the meta-statistical analysis of MAP-related VOCs derived from the three studies with the VOCs assigned to the core-profile highlighted in bold letters.

Chemical class	VOC	n		Std Mean*		Adjusted p-value^#^
		MAP	CV	MAP	CV	
**Aldehydes**	**2-Methyl-2-butenal**	80	20	0.16	0.28	<0.001
	2-Methylbutanal	120	30	0.24	0.51	<0.001
	3-Methylbutanal	120	30	0.16	0.49	<0.001
	**Benzaldehyde**	46	20	0.11	0.53	<0.001
	**Heptanal**	46	20	0.07	0.56	<0.001
	**Hexanal**	120	30	0.08	0.52	<0.001
**Carbohydrates**	**2,4-Dimethylheptane**	80	20	0.58	0.03	<0.001
	**4-Methyloctane**	80	20	0.68	0.11	<0.001
	**Heptane**	120	30	0.52	0.01	<0.001
	**Hexane**	120	30	0.58	0.13	<0.001
	**Methyl-cyclo-pentane**	46	20	0.78	0.36	<0.001
	**Octane**	120	30	0.58	0.01	<0.001
	**Pentane**	120	30	0.67	0.02	<0.001
	**2-Methylpentene**	80	20	0.63	0.33	= 0.004
	**Benzene**	80	20	0.6	0.12	<0.001
**Alcohols**	**2-Methylbutanol**	46	20	0.61	0.00	<0.001
	**3-Methylbutanol**	46	20	0.52	0.00	<0.001
**Ester**	**Methyl-acetate**	80	20	0.26	0.00	<0.001
**Furans**	**2,3,5-Trimethylfuran**	46	20	0.8	0.11	<0.001
	**2-Ethylfuran**	120	30	0.62	0.36	<0.001
	**2-Pentylfuran**	80	20	0.5	0.16	<0.001
	**2-Propylfuran**	46	20	0.64	0.35	<0.001
	**3-Methylfuran**	80	20	0.52	0.08	<0.001
**Ketones**	**2,3-Butadione**	46	20	0.63	0.01	<0.001
	**2-Butanone**	120	30	0.67	0.23	<0.001
	**2-Pentanone**	46	20	0.79	0.3	<0.001
	**3-Octanone**	120	30	0.49	0.02	<0.001
	**3-Pentanone**	46	20	0.74	0.02	<0.001
	**Acetone**	120	30	0.65	0.15	<0.001
	**Methyl-isobutyl-ketone**	80	12	0.73	0.39	<0.001
**Variable**	2-Methylpropanal	120	30	0.3	0.35	0.32
	2,4-Dimethylheptene	80	20	0.33	0.02	0.03
	3-Octanol	46	20	0.42	0.19	0.19
	Hexanol	46	20	0.37	0.3	0.77
	2-Methylfuran	80	12	0.68	0.4	0.05
	Furan	120	30	0.49	0.4	0.19
	2-Heptanone	120	30	0.28	0.4	0.32
	2-Methylbutanenitrile	80	12	0.72	0.65	0.18
	Dimethyldisulfid	46	20	0.59	0.52	0.29

Bold: selected core-profile, n: number of measurements

*: Mean of standardized concentration values, CV: control vials

^#^: Benjamini-Hochberg FDR method.

Substances belonged to the chemical classes of aldehydes (n = 6), carbohydrates (n = 9), alcohols (n = 2), furans (n = 5), ketones (n = 7) and one ester. Twenty-five substances were identical to the profile of the intersection analysis. Three substances which indicated MAP-growth in the intersection analysis were confirmed to be significant for MAP by the meta-statistics, 2-Methylbutanenitrile, 2,4-Dimethylheptene and 2-Methylfuran. In contrast, the meta-statistical analysis revealed four substances being significant for MAP, which were measured in at least two of the studies but only significant in one of them, 2-Methylpentene, 2-Methylbutanol, 3-Methylbutanol and Methyl-isobutyl-ketone (S1 Table). Two substances, 2-Methylbutanal and 3-Methylbutanal, were significant for MAP according to the meta-statistical analysis, but showed opposite tendencies amongst the three studies (Fig 2, ↓↑). These compounds were excluded from further analyses. The remaining 28 VOCs with identical tendencies amongst the three studies were defined as the VOC core profile of MAP.

**Fig 3:**
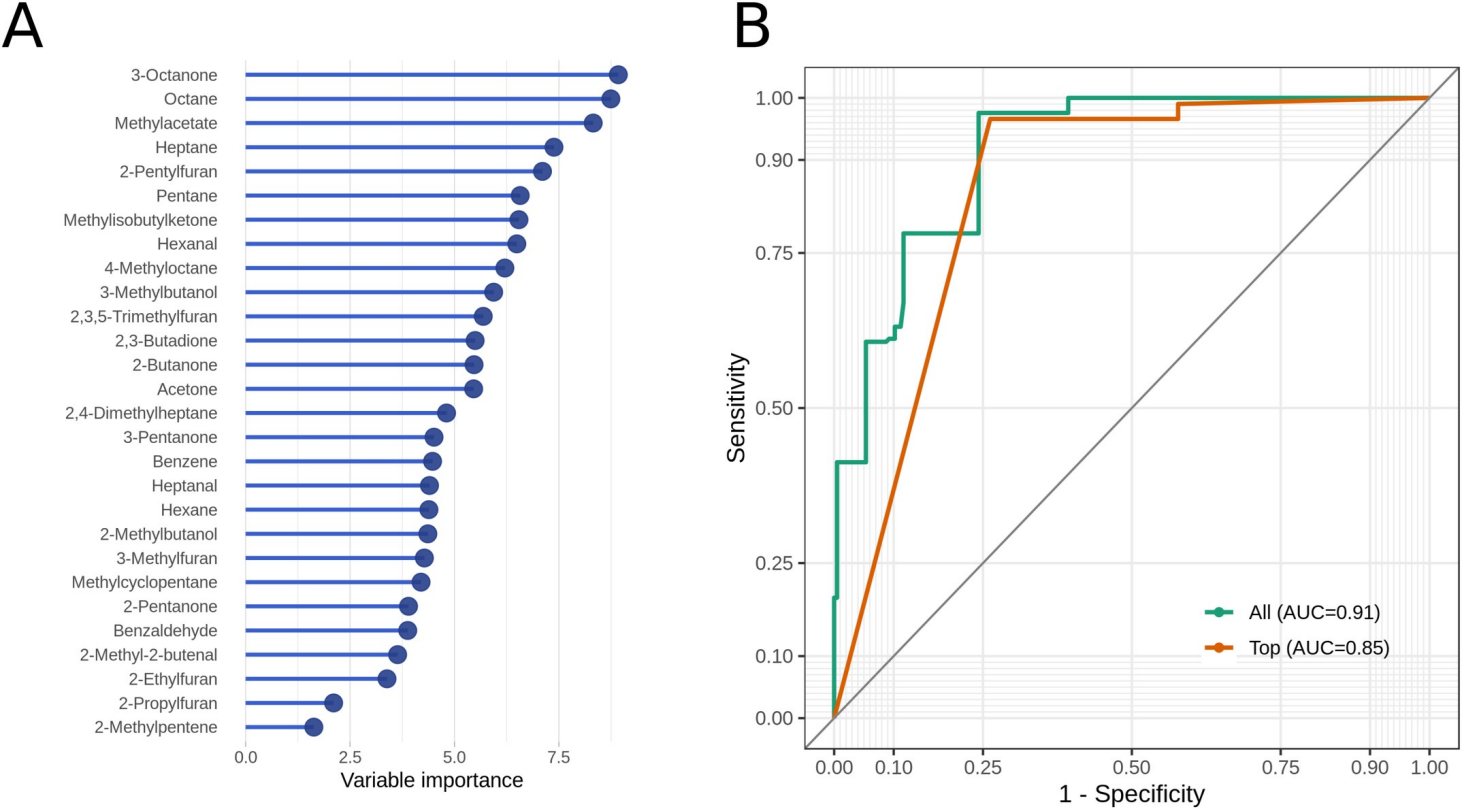
Random forest classification results. A: Variable importance plot of all relevant VOCs according to the Boruta feature selection. B: Receiver operating characteristic (ROC) curves of the cross-validated Random forest classification on test data. TOP represents classification for the single feature 3-Octanone, while ALL includes 28 previously selected VOCs. The diagonal line represents the baseline for random classification. AUC–area under the curve.

To test the capability of this VOC core profile to diagnose MAP status, we subjected it to Random forest classification. Using feature importance calculation by Boruta the 28 substances were ranked from highest to lowest importance. The most important substance was 3-Octanone, followed by Octane, Methyl-acetate, Heptane and 2-Pentylfuran (Fig 3A). None of the VOCs were declared non-relevant, therefore, classification was run with the entire core profile. Particularly, we performed cross-validated classification on all pairs of data sets, as described in Methods.

The accuracy of the classification was visualized by ROC curves for all features and for the top-ranked feature 3-Octanone, respectively (Fig 3B). Generally, very good results were achieved, with area under curve (AUC) values of 0.91 for all features and 0.85 for 3-Octanone.

## Discussion

This is the first time that a specific core volatilome for a mycobacterial species was defined. The core-profile was extracted from the results of three independent studies, which were performed in different years, by different experimenters and with variable experimental set-up [14, 19, 20]. Several statistical analyses were applied to identify a robust VOC-profile indicating MAP-growth. Consequently, we now propose a core-profile of 28 substances. These VOCs result from a meta-statistical analysis, excluding substances that showed opposite tendencies of relative change in the different studies. The first approach to compare the three studies was a set-based intersection analysis that took the defined VOC-profiles of each study into account. This mode of comparison considered the overlap of all study sets. The second approach combined all measurements of all studies in a meta-statistical analysis. The resulting larger sample sizes are beneficial for the statistical power, however they may also be affected by inter-study variance. To minimize the influence of such effects on the results, standardization and rank-based hypothesis tests were applied. The proposed VOC core profile appears to be robust, because it could be reproduced by different statistical approaches. Robustness of the VOC core profile reflects its reproducibility despite methodological variability, which is a pre-requisite for further development towards diagnostic application.

The proposed VOC core profile can be seen as the VOC signature of MAP, which does not consist of one individual biomarker but takes different substances with different trends in concentration relative to a control sample into account. This is comparable to the concept of clinical and pathophysiological examination of patients, where trends in the alteration (decrease or increase) of a variety of biomarkers relative to healthy subjects form the basis of diagnosis. In contrast, a concept of one or few species specific biomarkers was followed by others [25].

The experimental set-up of the three studies varied in different aspects: batch and age of medium, inoculum, duration of incubation and pre-concentration technique (Table 2). This could explain the occurrence of VOCs with different tendencies in concentration in the individual studies. The most important variable in this respect seemed to be bacterial density, which varied depending on the inoculum (Table 2) and increased during the incubation period. Due to different stages of bacterial growth after different periods of incubation, quantitative and qualitative changes in the VOC-profiles above bacteria are to be expected [10, 14–16, 18]. This was shown previously for aldehydes, which increased above vials inoculated with very low bacterial density before they decreased up to below the limit of detection [14]. The four aldehydes included in the MAP-core-profile decreased above MAP cultures in all three studies. Nonetheless, it was demonstrated previously that specific VOC patterns can be detected already in early stages of MAP growth [14]. Therefore, VOC analysis is considered a powerful tool to accelerate cultural detection of MAP infection.

Different techniques of pre-concentration were used in the three studies (Table 2). The limit of detection of several substances was shown to depend on the pre-concentration technique [26, 27]. Therefore, some alkanes such as Methyl-cyclo-pentane could not be detected in Study 1. Other substances, which were assigned to the MAP-indicative profile in at least one of the studies were not detectable via SPME at all [27], i. e. alcohols (3-Octanol), 2-Methyl-2-butenal, Heptanal, Benzene, 3-Methylfuran and 2-Butanone, others were not captured by NTME, for example Methyl-acetate and 2-Methylfuran [27]. In addition, the concentration levels of individual substances varied amongst the three studies. This effect may be due to different binding affinity of substances to the packing materials used in the pre-concentration devices as well as different desorption effects [28]. However, these sources of variability can be overcome by standardized sampling and analysis protocols.

Different batches and the age of the medium have to be considered further sources of variability in VOC emissions. Herrold´s Egg Yolk medium is not a purely synthetic medium but contains egg yolk that may vary in composition between batches resulting in modifications in the emitted VOCs. Furthermore, variation in age of the medium at the time point of inoculation needs also to be taken into account. Despite none of the media used was expired, aging may as well have caused alterations in media composition and consequentially in VOC emission. The two latter factors, cannot be circumvented and will always be a source of variability if VOC analysis will find its way in routine laboratory practice.

The specificity of the VOC profile is mandatory for the diagnostic application of VOC analysis. However, the VOC core profile of MAP defined in this study covers also substances that were considered as indicating mycobacterial growth in general in Study 3 [20]. This concerns 2-Methylbutanol, Pentane, Heptane, Octane, 2,3-Butadione, 3-Pentanone and 3-Octanone, which all increased over cultures. Evidently, these substances originate from common mycobacterial metabolic pathways, although these pathways still have to be revealed. Otherwise, the VOC core profile covers also four substances which have been defined as MAP specific in Study 3: 3-Pentanone, 2-Propylfuran, 2,3,5-Trimethylfuran and Pentane [20]. Specificity of the VOC signature of MAP is not only defined by the compounds included but also by the relative change of the concentration of these substances above MAP cultures. For example 3-Pentanone increased above all mycobacteria, but above MAP it reached the highest concentrations compared to all other 12 mycobacteria species [20]. Thus, the magnitude of change in concentration compared to controls may be more informative than absolute values.

The suitability of an analytic tool for diagnostic application depends on the accuracy of its classification of real samples. Among other enhanced statistical methods, Random forests are suitable for analyzing patterns in VOC data sets because they consider multiple compounds of a sample simultaneously [29]. Random forest in general represents a stable and robust classification method that is characterized particularly by its low number of parameters and strong performance in constructing a valid classification model [30]. In this study, Random forests were utilized in two ways: first, to calculate significant importance scores and ranking of the compounds (feature selection) and second, to classify MAP versus control samples based on the VOC core profile. Feature selection is an important part of the classification process because the associated score allows to rank substances and to distinguish VOCs that are more or less essential for the classification outcome. Recently, various feature selection approaches were compared regarding their performance, stability and relevance of the resulting feature set and the Boruta algorithm appeared as one of the best [31]. Consequently, the Boruta strategy was applied to select important variables against a randomized control. As a result, all 28 variables were considered relevant, although with varying degree of importance. Classification was performed by a multivariate approach using all 28 compounds, in comparison to a univariate approach including only the top-ranked compound, 3-Octanone.

Interestingly, already the top-univariate model can achieve high discrimination between MAP and control samples (AUC 0.85). This was not surprising looking at the relative changes shown in Fig 2 with value distributions of 3-Octanone consistently larger than control in all three data sets. However, the multivariate model resulted in further improvement of classification to an AUC value of 0.91, which was mainly due to a decrease in false positive results (Fig 3B). Therefore, the multivariate approach seems to be advantageous for proper classification.

The entire classification approach was cross-validated using multiple data sets that were derived from different experimental set-ups. This ensured that the reported results represent more accurate models and therefore better estimations of the classification performance, which is the major benefit of training on several data-sets.

Feces and different tissues are natural diagnostic samples for cultural isolation of MAP. No data are available about VOCs emitted by these matrices and the gut microbiota during cultivation. Interference of these VOCs with the MAP specific VOC profile is likely and has to be addressed as a next step in the development towards diagnostic application.

## Conclusions

This is the first comparative data analysis of several individual studies targeting a VOC-profile of a specific bacterium, i. e. MAP. We were able to define a VOC core profile indicating MAP-growth that consisted of 28 substances. Concentrations of four compounds decreased above MAP and 24 increased. Even though the three studies were carried out with slight differences in methodology, the robustness of the defined core-volatilome is underlined by the outcome of two statistical analyses. Using the VOC core profile classification of MAP samples was possible in a testing scenario of different experimental platforms and datasets.

The discriminatory power of this tool for the characterization of natural diagnostic samples, in particular its diagnostic specificity, has to be investigated in future studies.

## Supporting information

S1 Table Substances that could be measured in at least two studies. Median, first and third quartile of VOC concentration (ppbV) above MAP and above pure media (control vials).
(DOCX)

S2 Table Relative change of VOC concentration of MAP cultures compared to control vials. (DOCX)
