# Supplementary material for: Core profile of volatile organic compounds related to growth of Mycobacterium avium subspecies paratuberculosis – A comparative extract of three independent studies
Source: PLoS One. 2019 Aug 15;14(8):e0221031. doi: 10.1371/journal.pone.0221031 (PMC6695172; doi:10.1371/journal.pone.0221031)
Supplement: S1 Table — Median, first and third quartile of VOC concentration (ppbV) above MAP and above pure media (control vials). (DOCX) [file pone.0221031.s001.docx]

**S1_Table.docx**

**S1 Table**: **Substances that could be measured in at least two studies**. Median, first and third quartile of VOC concentration (ppbV) above MAP and above pure media (control vials).

| **Study** | **1 [20]** | | | | | | | **2 [14]** | | | | | | | | **3 [19]** | | | | | | | |
| --- | --- | --- | --- | --- | --- | --- | --- | --- | --- | --- | --- | --- | --- | --- | --- | --- | --- | --- | --- | --- | --- | --- | --- |
|  | **MAP** | | |  | **control vials** | | | **MAP** | | |  | **control vials** | | | **MAP** | | | |  | **control vials** | | |  |
| **Dilutions** | **10^0^, 10^-2^, 10^-4^, 10^-6^** | | |  | **none** | | | **10^0^, 10^-2^, 10^-4^, 10^-6^** | | |  | **none** | | | **10^0^** | | | |  | **none** | | |  |
| **Period of incubation** | **6 weeks** | | |  | **6 weeks** | | | **6 weeks** | | |  | **6 weeks** | | | **4 weeks** | | | |  | **4 weeks** | | |  |
| VOC (ppbV) | Q1 | **Median** | Q3 |  | Q1 | **Median** | Q3 | Q1 | **Median** | Q3 |  | Q1 | **Median** | Q3 | Q1 | | **Median** | Q3 |  | Q1 | **Median** | Q3 |  |
| 2-Methylbutanol | . | . | . |  | . | . | . | 180.84 | **418.49** | 827.70 | ***** | 1.45 | **1.45** | 1.45 | 76.25 | | **86.77** | 95.64 |  | 0.57 | **0.57** | 0.57 |  |
| 3-Methylbutanol | . | . | . |  | . | . | . | 450.39 | **1111.23** | 2040.51 | ***** | 1.96 | **1.96** | 1.96 | 132.48 | | **186.21** | 203.04 |  | 1.38 | **1.38** | 1.38 |  |
| 3-Octanol | . | **.** | . |  | . | **.** | . | 20.85 | **36.57** | 46.34 | ***** | 14.32 | **15.23** | 16.60 | 19.29 | | **25.83** | 30.56 | ***** | 1.66 | **1.66** | 1.66 |  |
| Hexanol | . | . | . |  | . | . | . | 9.78 | **16.67** | 25.89 | ***** | 1.30 | **1.39** | 1.67 | 0.83 | | **0.83** | 0.83 |  | 1.00 | **1.63** | 2.42 |  |
| 2-Methyl-2-butenal | 42.80 | **63.26** | 94.19 | ***** | 165.90 | **170.36** | 174.83 | 2.45 | **2.45** | 2.45 |  | 19.67 | **20.38** | 21.03 | . | | **.** | . |  | . | **.** | . |  |
| 2-Methylbutanal | 750.39 | **929.40** | 1306.60 | ***** | 563.58 | **575.14** | 586.70 | 262.64 | **500.32** | 779.55 | ***** | 917.99 | **950.35** | 1003.46 | 29.09 | | **29.40** | 30.07 | ***** | 1034.97 | **1133.50** | 1268.89 |  |
| 2-Methylpropanal | 468.71 | **577.61** | 651.95 | ***** | 321.00 | **324.87** | 328.73 | 1981.74 | **2305.64** | 2802.60 | ***** | 1399.69 | **1564.92** | 1666.76 | 444.88 | | **461.27** | 473.13 | ***** | 1018.35 | **1206.01** | 1436.02 |  |
| 3-Methylbutanal | 975.87 | **1316.73** | 1542.24 | ***** | 950.66 | **969.53** | 988.40 | 357.02 | **677.98** | 926.42 | ***** | 1569.91 | **1601.98** | 1700.82 | 9.49 | | **10.74** | 11.25 | ***** | 462.47 | **502.46** | 565.95 |  |
| Benzaldehyde | . | **.** | . |  | . | **.** | . | 4.89 | **5.44** | 7.27 | ***** | 44.21 | **47.17** | 50.65 | 3.36 | | **3.36** | 3.36 | ***** | 38.82 | **42.70** | 55.51 |  |
| Heptanal | . | **.** | . |  | . | **.** | . | 0.33 | **0.33** | 0.33 | ***** | 1.04 | **1.12** | 1.26 | 3.51 | | **3.51** | 3.51 | ***** | 7.53 | **7.91** | 9.66 |  |
| Hexanal | 3.89 | **7.33** | 10.19 | ***** | 33.75 | **35.02** | 36.28 | 27.87 | **39.32** | 46.69 | ***** | 1243.54 | **1293.97** | 1381.95 | 22.75 | | **22.75** | 22.75 | ***** | 759.45 | **833.30** | 1007.38 |  |
| 2,3,5-Trimethlfuran | . | **.** | . |  | . | **.** | . | 19.64 | **22.23** | 23.87 | ***** | 1.50 | **1.71** | 1.89 | 10.81 | | **11.61** | 12.67 | ***** | 1.48 | **1.58** | 1.80 |  |
| 2-Ethylfuran | 347.37 | **478.33** | 650.28 | ***** | 13.33 | **13.62** | 13.91 | 815.60 | **1053.07** | 1254.32 | ***** | 425.24 | **457.92** | 488.53 | 445.67 | | **494.44** | 524.13 |  | 382.30 | **401.31** | 469.47 |  |
| 2-Methylfuran | 116.40 | **159.37** | 222.20 | ***** | 37.08 | **37.70** | 38.32 | . | **.** | . |  | . | **.** | . | 355.19 | | **369.40** | 408.03 | ***** | 253.12 | **285.84** | 351.69 |  |
| 2-Pentylfuran | 2073.28 | **3664.69** | 4896.91 | ***** | 41.22 | **42.85** | 44.48 | 87.25 | **141.16** | 163.44 |  | 60.04 | **66.09** | 68.81 | . | | **.** | . |  | . | **.** | . |  |
| 2-Propylfuran | . | **.** | . |  | . | **.** | . | 8.13 | **15.50** | 19.94 | ***** | 5.46 | **5.78** | 6.37 | 4.68 | | **4.99** | 5.97 | ***** | 2.78 | **2.91** | 3.40 |  |
| 3-Methylfuran | 20.77 | **27.67** | 35.76 | ***** | 5.51 | **5.62** | 5.74 | 422.57 | **673.71** | 871.20 | ***** | 24.35 | **26.83** | 28.28 | 55.48 | | **65.45** | 69.82 |  | 21.11 | **22.50** | 28.04 |  |
| Furan | 57.69 | **63.44** | 75.01 | ***** | 18.71 | **19.28** | 19.84 | 285.53 | **411.76** | 491.51 | ***** | 191.64 | **221.10** | 243.46 | 52.06 | | **86.45** | 104.98 |  | 204.99 | **217.72** | 245.91 |  |
| 2,3-Butadione | . | **.** | . |  | . | **.** | . | 138.47 | **444.40** | 561.73 | ***** | 4.82 | **4.82** | 4.95 | 190.53 | | **204.83** | 213.57 | ***** | 3.97 | **5.20** | 5.51 |  |
| 2-Butanone | 67.60 | **88.13** | 97.18 | ***** | 24.59 | **25.18** | 25.77 | 682.08 | **791.22** | 883.96 | ***** | 235.33 | **237.79** | 245.39 | 385.01 | | **402.35** | 420.02 | ***** | 125.92 | **142.03** | 169.95 |  |
| 2-Heptanone | 271.29 | **479.27** | 639.61 | ***** | 30.32 | **31.69** | 33.06 | 13.72 | **16.97** | 19.68 | ***** | 34.62 | **37.55** | 42.54 | 3.84 | | **4.27** | 4.56 | ***** | 8.09 | **9.08** | 12.18 |  |
| 2-Pentanone | . | **.** | . |  | . | **.** | . | 32.04 | **37.53** | 40.96 | ***** | 8.74 | **9.09** | 10.11 | 14.74 | | **14.84** | 16.31 | ***** | 5.42 | **6.12** | 7.82 |  |
| 3-Octanone | 876.07 | **1268.31** | 1888.60 | ***** | 75.64 | **75.64** | 75.64 | 23.55 | **28.22** | 47.61 | ***** | 0.77 | **0.77** | 0.77 | 16.05 | | **16.23** | 19.20 | ***** | 0.89 | **0.89** | 0.89 |  |
| 3-Pentanone | . | **.** | . |  | . | **.** | . | 70.16 | **101.00** | 108.56 | ***** | 1.94 | **2.38** | 2.68 | 92.59 | | **104.02** | 110.60 | ***** | 1.47 | **1.66** | 1.69 |  |
| Acetone | 340.64 | **415.90** | 456.86 | ***** | 60.86 | **62.09** | 63.32 | 9787.75 | **10488.94** | 13083.56 | ***** | 3866.69 | **4019.93** | 4396.15 | 11918.44 | | **13025.55** | 14504.16 | ***** | 2105.59 | **2293.62** | 2479.01 |  |
| Methyl-isobutyl-ketone | 21.68 | 25.74 | 30.10 | ***** | 14.48 | 14.81 | 15.13 | . | . | . |  | . | . | . | 3.54 | | 4.09 | 4.48 |  | 1.69 | 1.82 | 1.95 |  |
| Methyl-acetate | 33.64 | **37.92** | 100.81 | ***** | 3.09 | **3.32** | 3.54 | 69.00 | **242.05** | 320.17 | ***** | 5.44 | **5.44** | 5.44 | . | | **.** | . |  | . | **.** | . |  |
| 2-Methylbutanenitrile | 21.24 | 27.03 | 30.05 | * | 20.98 | 21.27 | 21.56 | 0.97 | 0.97 | 0.97 | * | 11.32 | 11.57 | 11.98 | 4.67 | | 5.27 | 5.88 |  | 4.35 | 4.70 | 5.48 |  |
| Dimethyldisulfid | 140.94 | 168.75 | 263.71 | * | 60.05 | 61.44 | 62.83 | 15.44 | 18.23 | 32.95 |  | 22.14 | 23.40 | 25.13 | 24.66 | | 26.78 | 28.17 |  | 20.43 | 21.04 | 23.33 |  |
| 2,4-Dimethylheptane | 577.51 | 676.93 | 737.38 | * | 49.52 | 51.86 | 54.19 | 2.16 | 6.12 | 6.89 | * | 2.16 | 2.16 | 2.16 | . | | . | . |  | . | . | . |  |
| 4-Methyloctane | 270.66 | 316.64 | 353.64 | * | 27.78 | 28.62 | 29.46 | 5.01 | 5.82 | 6.63 | * | 1.76 | 1.76 | 2.34 | . | | . | . |  | . | . | . |  |
| Heptane | 124.45 | 153.47 | 173.75 | * | 5.80 | 5.85 | 5.90 | 445.80 | 582.50 | 714.64 | * | 4.24 | 4.24 | 4.24 | 54.70 | | 64.06 | 74.34 | * | 0.25 | 0.25 | 0.25 |  |
| Hexane | 59.43 | 77.77 | 88.48 | * | 26.08 | 26.81 | 27.54 | 2929.34 | 3476.58 | 4046.74 | * | 420.56 | 462.43 | 500.40 | 47.06 | | 48.90 | 53.19 | * | 9.51 | 10.36 | 10.56 |  |
| Methyl-cyclo-pentane | . | . | . |  | . | . | . | 3.59 | 4.63 | 5.23 | * | 1.32 | 1.41 | 1.55 | 4.99 | | 5.28 | 5.49 | * | 2.68 | 2.89 | 3.00 |  |
| Octane | 54.31 | 78.46 | 89.75 | * | 3.62 | 4.42 | 5.22 | 219.07 | 274.56 | 356.54 | * | 4.02 | 4.02 | 4.02 | 24.08 | | 26.00 | 44.28 | * | 0.56 | 0.56 | 0.56 |  |
| Pentane | 1351.89 | 1679.97 | 2134.96 | * | 106.74 | 111.93 | 117.13 | 13323.78 | 38440.38 | 48398.95 | * | 688.86 | 723.46 | 828.86 | 54548.80 | | 61477.87 | 65871.91 | * | 507.49 | 633.58 | 779.26 |  |
| 2,4-Dimethylheptene | 512.92 | 606.63 | 661.53 | * | 42.88 | 43.94 | 45.00 | 2.16 | 12.30 | 15.70 | * | 2.16 | 2.16 | 2.16 | . | | . | . |  | . | . | . |  |
| 2-Methylpentene | 406.03 | 581.88 | 703.38 | * | 75.17 | 78.14 | 81.12 | 30.11 | 35.97 | 41.22 |  | 25.69 | 31.39 | 35.75 | . | | . | . |  | . | . | . |  |
| Benzene | 18.29 | 23.71 | 26.45 |  | 1.75 | 1.85 | 1.95 | 280.49 | 310.80 | 357.36 |  | 66.46 | 70.45 | 74.36 | . | | . | . |  | . | . | . |  |

MAP: *Mycobacterium avium* ssp. p*aratuberculosis,* Q1: percentile 0.25, Q3: percentile 0.75, *: p-value <0.05 evaluated by authors of the individual studies
