# Supplementary material for: Core profile of volatile organic compounds related to growth of Mycobacterium avium subspecies paratuberculosis – A comparative extract of three independent studies
Source: PLoS One. 2019 Aug 15;14(8):e0221031. doi: 10.1371/journal.pone.0221031 (PMC6695172; doi:10.1371/journal.pone.0221031)
Supplement: S2 Table — (DOCX) [file pone.0221031.s002.docx]

**S2_Table.docx**

**S2 Table: Relative change of VOC concentration above MAP cultures compared to control vials.**

|  | Study 1 | Study 2 | Study 3 |
| --- | --- | --- | --- |
| VOCs | Median | Median | Median |
| **2-Methyl-2-butenal** | 0.37 | 0.12 |  |
| *2-Methylbutanal* | 1.62 | 0.53 | 0.03 |
| *2-Methylpropanal* | 1.78 | 1.47 | 0.38 |
| *3-Methylbutanal* | 1.36 | 0.42 | 0.02 |
| **Benzaldehyde** |  | 0.12 | 0.08 |
| **Heptanal** |  | 0.3 | 0.44 |
| **Hexanal** | 0.21 | 0.03 | 0.03 |
| 2,4-Dimethylheptane | 13.05 | 2.83 |  |
| 4-Methyloctane | 11.06 | 3.31 |  |
| Heptane | 26.23 | 137.38 | 256.25 |
| **Hexane** | 2.9 | 7.52 | 4.72 |
| **Methyl-cyclo-pentane** |  | 3.29 | 1.83 |
| **Octane** | 17.75 | 68.3 | 46.42 |
| **Pentane** | 15.01 | 53.13 | 97.03 |
| 2,4-Dimethylheptene | 13.8 | 5.7 |  |
| Benzene | 12.82 | 4.41 |  |
| *3-Octanol* |  | 0.02 | 15.56 |
| **Methyl-acetate** | 11.44 | 44.49 |  |
| 2,3,5-Trimethylfuran |  | 13.01 | 7.35 |
| 2-Ethylfuran | 35.12 | 2.3 | 1.23 |
| **2-Methylfuran** | 4.23 |  | 1.29 |
| 2-Pentylfuran | 85.52 | 2.14 |  |
| **2-Propylfuran** |  | 2.68 | 1.71 |
| 3-Methylfuran | 4.92 | 25.11 | 2.91 |
| Furan | 3.29 | 1.86 | 0.4 |
| **2,3-Butadione** |  | 92.2 | 39.38 |
| **2-Butanone** | 3.5 | 3.33 | 2.83 |
| *2-Heptanone* | 15.12 | 0.45 | 0.47 |
| **2-Pentanone** |  | 4.13 | 2.43 |
| **3-Octanone** | 16.77 | 36.65 | 18.23 |
| **3-Pentanone** |  | 42.42 | 62.72 |
| **Acetone** | 6.7 | 2.61 | 5.68 |
| **2-Methylbutanenitrile** | 1.27 |  | 1.12 |

**Bold**: relative change lies in the same range of values for all available data, *italic*: different tendencies amongst studies
